# Current management of papillary thyroid microcarcinoma in Canada

**DOI:** 10.1186/s40463-014-0032-8

**Published:** 2014-08-14

**Authors:** Mazin Merdad, Antoine Eskander, John De Almeida, Jeremy Freeman, Lorne Rotstein, Shereen Ezzat, Anna M Sawka, David P Goldstein

**Affiliations:** 1 Department of Otolaryngology ¿ Head and Neck Surgery, University Health Network, Princess Margaret Hospital, University of Toronto, Rm 3-952, 610 University Ave, Toronto M5G 2M9, Ontario, Canada; 2 Department of Otolaryngology ¿ Head and Neck Surgery, King Abdulaziz University, Jeddah, Saudi Arabia; 3 Department of Otolaryngology ¿ Head and Neck Surgery, Mount Sinai Hospital, University of Toronto, Toronto, Ontario, Canada; 4 Department of General Surgery, Toronto General Hospital, University of Toronto, Toronto, Ontario, Canada; 5 Division of Endocrinology, Department of Medicine, University Health Network, University of Toronto, Toronto, Ontario, Canada

**Keywords:** Thyroid cancer, Microcarcinoma, Papillary thyroid cancer, Well-differentiated thyroid cancer, Survey, Questionnaire

## Abstract

**Introduction:**

The detection of papillary thyroid microcarcinoma (PTMC) is on the rise and its optimal management remains controversial. Our aim was to determine the current self-reported management of PTMC amongst Canadian otolaryngologist-head and neck surgeons (OHNS) and endocrinologists and to identify factors influencing their management decisions.

**Methods:**

A nine item web-based questionnaire was distributed to Canadian OHNS and endocrinologists. The three main domains were demographics, current management of PTMC scenarios, and factors influencing the decisions.

**Results:**

One hundred and thirteen OHNS and endocrinologists completed the survey. Respondents were closely divided between recommending hemithyroidectomy (47%) or total thyroidectomy (43%) for a newly diagnosed PTMC in a low risk patient. Observation was the preferred method for managing PTMC detected incidentally after hemithyroidectomy (76%). Respondents chose more aggressive treatment for male patients compared to female patients. A positive history of thyroid cancer or previous radiation exposure was the most important factor influencing the management of PTMC.

**Conclusion:**

The current practices of Canadian OHNS and endocrinologist largely coincide with available guidelines. The slight variation in practice might be explained by the opposing evidence supporting different management options. Given the dramatic increase in the incidence of PTMC we suggest future guidelines address the management of PTMC independently.

## Introduction

Papillary thyroid cancer (PTC) incidence rates are on the rise largely due to the increased detection rate attributed to improvements in the quality, availability, and utilization of medical imaging [1]. At present, the majority of newly diagnosed malignant thyroid nodules are 2 cm or less [2],[3]. Papillary thyroid mircocarcinomas (PTMC), defined as cancers less than 1 cm in maximum diameter, are currently the most prevalent PTC's accounting for an estimated 39-48% of all PTC's [3]¿[6]. The prognosis of PTMC remains excellent with disease specific mortality well under 1% [3],[7]¿[9].

Controversy remains around the extent of surgery required for T1 and T2 PTCs. The latest thyroid nodule management guidelines published by the European Thyroid Association (ETA; 2006), British Thyroid Association (BTA; 2007), and American Thyroid Association (ATA; 2009) address the management of PTMC within the context of managing PTC [10]¿[12]. Recent long-term survival data from high-volume centers support a less aggressive management of PTMC than those advocated by existing guidelines [13],[14].

The primary objective of this study was to determine the current self-reported management of PTMC amongst Canadian otolaryngologists-head and neck surgeons (OHNS) and endocrinologists and to identify important factors affecting their management decisions. A secondary objective was to compare the treatment recommendations to the currently available management guidelines.

## Methods

A nine-item questionnaire was developed to determine the current practice of OHNS and endocrinologists in managing PTMC and assessing the importance of several factors that may influence these management decisions. The survey was distributed as a web-based questionnaire to active members of the Canadian Society of Otolaryngology- Head and Neck Surgery (CSO-HNS; 468 members) and the Canadian Society of Endocrinology and Metabolism (CSEM; 390 members). The initial invitation e-mail was sent out February 2013 with a brief cover letter and a link to our survey. A follow-up questionnaire was sent 4 weeks later. An attempt was made to distribute the survey to general surgeons through their Canadian society, however due to logistics, the society was unable to distribute the survey through private E-mail addresses.

**Figure 1:**
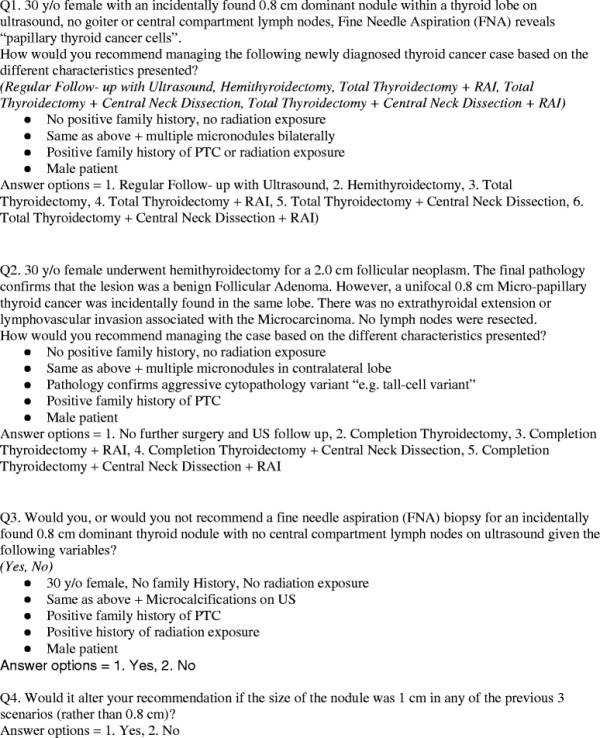
Management scenario questions (Part B).

The questionnaire was developed using a modified Delphi method, which was reviewed by experts in the field of endocrinology and endocrine surgery to ensure that the collected information met the objectives (face validity). Before widespread dissemination, the survey was piloted on five surgeons to confirm that the survey was easy to understand and respond to (cognitive testing). The survey was comprised of three domains; demographic (Part A: 4 questions), current management of PTMC (Part B: 4 questions), and factors influencing these decisions (Part C: 9 questions). To determine current practice management (Part B), case scenarios were presented with several management options. To assess the importance of factors governing these decisions, factors were enumerated and a 5-point Likert-scale was used to assess importance of each of these factors. Figure 1 displays the survey¿s management scenario questions (Part B).

Statistical analysis was performed using IBM SPSS statistical software version 20.0 (IBM, Armonk, NY, USA). Categorical and ordinal variables were compared using *?*^2^ or Mann¿Whitney U tests. Statistical significance was considered present at P values?<?0.05.

## Results

### Response rate

A total of 113 OHNS and endocrinologists completed the survey. The response rate for the OHNS was 12% (50/409) and for endocrinologists 16% (63/390).

### Demographic results (Part A)

Approximately 56% of the responding OHNS were considered high volume specialists (performing?>?40 thyroid surgeries per year). Twenty-two percent of endocrinologists that responded had >25% of their practice dedicated to managing thyroid disease. Table 1 summarizes the demographics of the survey participants (age, practice type, volume).

**Table 1:** Demographics of participants (n?=?113)

**Variable**	**Category**	**Frequency (%)**
**Age in years**	25-34	21 (18.6%)
	35-44	37 (32.7%)
	45-54	30 (26.5%)
	55-64	12 (10.6%)
	65-74	6 (5.3%)
	75 or older	7 (6.2%)
**Practice type**	University/academic	63 (55.8%)
	Community or private	36 (31.9%)
	Both	14 (12.4%)
**Specialty**	Otolaryngology - Head & Neck Surgery	50 (44.2%)
	Endocrinology	63 (55.8%)
**OHNS average surgeries performed a year (n = 50)**	Does not perform thyroid surgeries	2 (4%)
	< 10	2 (4%)
	10 to 19	6 (12%)
	20 to 39	12 (24%)
	40 to 79	16 (32%)
	> 80	12 (24%)
**Endocrinologist percentage of practice devoted to managing thyroid neoplasm patients (n = 63)**	< 10%	17 (27%)
	11 to 25%	32 (51%)
	26 to 50%	8 (13%)
	51 to 75%	5 (8%)
	> 75%	1 (1.6%)

### Current management of PTMC (Part B)

**Figure 2:**
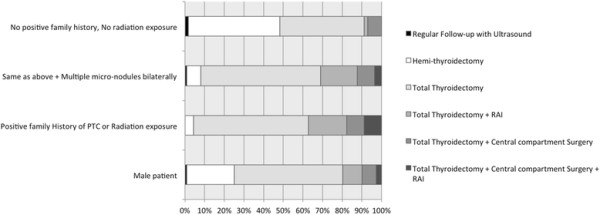
**Case #1 Responses.** Legend: Response to clinical scenario #1 (30 y/o female with an incidentally found 0.8 cm dominant nodule within a thyroid lobe on ultrasound, no goiter or central compartment lymph nodes, Fine Needle Aspiration (FNA) reveals ¿papillary thyroid cancer cells).

Figure 2 displays the responses for the first clinical scenario (newly diagnosed 0.8 cm with biopsy positive PTC cells). Without the presence of any risk factors, respondents were almost equally divided between recommending hemithyroidectomy (47%) or total thyroidectomy without central neck dissection (CND)/radioactive iodine (RAI) (43%). Only 2% recommended regular follow up with ultrasound without any surgical intervention. The recommendations were more aggressive in the presence of thyroid multi-nodularity, positive family history, or radiation exposure with nearly 60% of respondents recommending total thyroidectomy, and an additional 37% recommending total thyroidectomy plus RAI and/or CND. In the case of a male patient with the same history, 55% recommended a total thyroidectomy, and 20% total thyroidectomy plus RAI and/or CND. Respondents recommended a more aggressive form of treatment for male patients compared to female patients (p?=?<0.001). There were no statistically significant differences in recommendations by OHNS and endocrinologists in each of the different changes to the scenario except for the male patient variable. A significantly greater proportion of endocrinologists recommended total thyroidectomy in male patients than OHNS (82% vs. 65%, respectively) (p?=?0.01).

**Figure 3:**
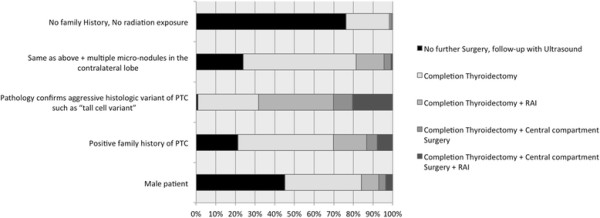
**Case #2 Responses.** Legend: Response to clinical scenario #2 (30 y/o female underwent hemithyroidectomy for a 2.0 cm follicular neoplasm. The final pathology confirms that the lesion was a benign Follicular Adenoma. A unifocal 0.8 cm Micro­papillary thyroid cancer was incidentally found in the same lobe. There was no extrathyroidal extension or lympho-vascular invasion associated with the Microcarcinoma. No lymph nodes were resected).

Figure 3 summarizes the responses for the second clinical scenario (incidentally found 0.8 cm PTMC in a hemithyroidectomy performed on a female patient for a benign nodule). Three fourths of respondents (76%) recommended no further surgery and follow up with ultrasound while 22% recommended completion thyroidectomy. In contrast, respondents were divided in their recommendations for male patients with 45% recommending no further surgery and continued surveillance and 48% recommending completion thyroidectomy with or without RAI. The presence of multinodularity, aggressive pathology (e.g. tall cell) and positive family history led to a more aggressive treatment recommendation. There were no statistically significant differences in recommendations by OHNS and endocrinologists for each of the different variables (p?=?0.08).

**Figure 4:**
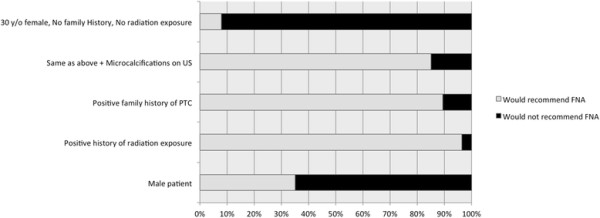
**Case #3 Responses.** Legend: Response to clinical scenario #3 (Would you, or would you not recommend a fine needle aspiration (FNA) biopsy for an incidentally found 0.8 cm dominant thyroid nodule with no central compartment lymph nodes on ultrasound).

Figure 4 summarizes responses for the third clinical scenario (when to perform a fine needle aspiration (FNA) on a 0.8 cm thyroid nodule). The majority of respondents (92%) recommended *against* performing FNA on a 0.8 cm nodule when the patient is a young female with a negative family and radiation history. The presence of microcalcifications on ultrasound, positive family history, or radiation exposure shifted the recommendations of the majority (85%, 90% and 97%, respectively) toward performing a FNA. For male patients, 35% recommended FNA compared to 65% recommending against. A larger proportion of respondents recommended FNA for male patients than for female patients with negative family and radiation history (p?<?0.001). There were no statistically significant differences in recommendations by OHNS and endocrinologists for each of the different variables (p?=?0.09).

In the fourth scenario (if a change in size, from 0.8 to 1 cm, in any of the three previous scenarios would have altered their management choice) around half the participants (54.5%) asserted that it would have altered their management. There were no statistically significant differences in recommendations by OHNS and endocrinologists (p?=?0.12).

### Factors influencing decision (Part C)

Table 2 presents the average rating (0?=?not important, 5?=?extremely important) of factors influencing respondents¿ decision when managing a case of newly diagnosed PTMC. Positive family history and/or radiation exposure were the most important factors influencing management with 96% of respondents rating these as important or extremely important factors. The ability to cure the patient with surgery followed by the low surgical morbidity associated with thyroid surgery were the second and third most important influencing factors, respectively. Comfort level observing a nodule that is confirmed to be malignant, patient preference, ease of patient follow up after total thyroidectomy were also identified as important factors influencing management decisions. The preference of the referring physician and fear of future litigation (malpractice) if the patient is observed leading to potentially adverse sequelae were reported as the two least important factors. There were no statistically significant differences in rating the importance of each factor by OHNS and endocrinologists.

**Table 2:** Factors influencing the management of PTMC (1 least important, 5?=?most important)

**Factor**	**Rating average**
**Patient preference**	3.61
**Patient risk factors (e.g. family history, radiation exposure)**	4.44
**Preference of the referring physician**	2.14
**Your comfort level observing a nodule that is confirmed to be malignant**	3.70
**Low surgical morbidity associated with thyroid surgery**	3.87
**Need for postoperative RAI**	3.10
**The ability to surgically cure the patient with surgery**	3.97
**Ease of patient follow up after total thyroidectomy surgery**	3.57
**Fear of future litigation (malpractice) if patient is observed and adverse sequelae occur**	2.64

## Discussion

Over the last two decades, the incidence of thyroid cancer has increased significantly, largely due to detection of subclinical disease [1]¿[3]. This has led to an increase in detection of the smallest tumors, particularly, microcarcinomas [3]¿[6]. This increased detection has had no impact on survival outcomes but has had a huge impact on patients and the health care system. Therefore, we sought to identify current practices in order to inform future guideline development, knowledge translation events, and quality improvement initiatives.

According to our survey results, the current treatment recommendations of endocrinologists and OHNS coincide with the available guidelines (Table 3) [10]¿[12]. Respondents were closely divided between recommending hemithyroidectomy or total thyroidectomy for a newly diagnosed PTMC in a low risk patient. The controversy and lack of consistent strong evidence supporting either treatment option is reflected in our respondents¿ recommendations where close to half (47%) recommended hemithyroidectomy and 43% recommended total thyroidectomy. Observation was the preferred method for managing PTMC detected incidentally after hemithyroidectomy. Three-fourths (76%) of respondents recommended only follow-up for a PTMC incidentally found on thyroid lobectomy while 24% recommended completion thyroidectomy. These findings are similar to the results from a recent survey of thyroid surgeons in the United States [15]. In their survey, Wu *et al.* found that after a hemithyroidectomy for PTMC, if there were no complicating factors, 70% of the respondents recommended no further surgery, while 30% believed that completion thyroidectomy was necessary [15]. Responses to the first two clinical scenarios in our survey demonstrate some inconsistencies amongst respondents. The majority of endocrinologists and OHNS would not recommend a completion thyroidectomy for patients with an incidental thyroid cancer, but a large proportion would recommend a total thyroidectomy upfront for patients with a confirmed cancerous subcentimeter lesion.

**Table 3:** Summary of ATA, BTA and ETA PTMC management recommendations

**ATA**		**BTA**	**ETA**
Investigate	> 5 mm if:	Can be managed by primary care physician if:	FNAC only if:
	1.suspicious US findings (microcalcifications; hypoechoic; increased nodular vascularity; infiltrative margins; taller than wide on transverse view).	1. non-palpable,	1. suspicious finding on US (solid hypoechoic with microcalcifications),
	2. history of head and neck radiation, or history of thyroid cancer in one or more first-degree relatives,	2. incidentally found,	2. personal history
	3. abnormal cervical lymph nodes	3. no concerning features	
Surgery	Lobectomy sufficient if: 1.no associated lymphadenopathy,	Lobectomy sufficient if:	Lobectomy sufficient if:
	2. no history of head and neck radiation or positive family history of thyroid cancer,	1. lymph node negative and followed by levothyroxine therapy,	1. no evidence or nodal or distant metastasis,
	3. low risk, unifocal, and intrathyroidal nodule	2. no multifocality,	2. no history of previous radiation exposure,
		3. no extrathyroidal spread,	3. multifocality,
		4. no family disease,	4. extrathyroidal extension,
		5. no metastasis,	5. vascular invasion,
		6. no vascular invasion,	6. unfavorable histology
		7. no contralateral disease	
RAI	Recommended if: distant metastasis or gross extrathyroidal extension.	Omit if :	No indication if:
	Not recommended if:	1. Unifocal,	1. complete surgery,
	1. unifocal and no high risk features,	2. N0 M0,	2. favorable histology,
	2. multifocal with all nodules?<?1 cm.	3. no extension beyond the thyroid,	3. N0 M0,
	Selective use if regional lymph node metastasis	4. favorable histology,	4. no extrathyroidal extension
		5. complete surgery	

In the scenario of a preoperatively diagnosed PTMC, the primary treatment options include the more extensively studied options of hemithyroidectomy and total thyroidectomy with or without postoperative RAI and the less established option of close observation [8],[13],[14],[16]¿[18]. Less than 2% of respondents in our survey recommended observation for a newly diagnosed PTMC on FNA whether or not the patient had any complicating factors. As far as we are aware, there is currently one observational study regarding the management of PTMC with close observation and no surgery. As more observational data get published, closely observing certain PTMCs might become a valid and safe option [8].

Male gender was the only added variable to the scenarios with consistent and significant deviation from the guidelines, with both OHNS and endocrinologists recommending a more aggressive treatment option for male patients compared to female patients. This is not supported by strong evidence or guideline recommendations [10]¿[12].

The exact size of the nodule mattered significantly to respondents. Nodules 1 cm in their largest diameter would have been treated differently than 0.8 cm nodules when all variables were held constant. To the best of our knowledge, there are no randomized trials comparing long term outcomes of PTMC (T1a) and small PTC?<?2 cm (T1b). This is likely due to difficulty accruing a large enough sample size to answer such a question. However, Sturgeon and colleagues reviewed the literature as well as retrospective data from major cancer registries showed a survival advantage for T1b patients who underwent total thyroidectomy compared to hemithyroidectomy. There was no survival advantage with the more extensive surgery in the T1a group [13],[16],[19].

The shift in management recommendations by our respondents as a result of a slight difference in size of the presented nodule, from 0.8 to 1.0 cm also highlights the importance of measuring nodule *volume* rather than maximum diameter to decrease the inter- and intra-observer variation in measurement [20]. Volumetric measurements may also assist in defining significant nodular growth [10].

Family history of thyroid cancer and/or previous radiation exposure were the most important factors influencing the management of PTMC amongst our respondents. The ability to cure the patient with surgery, and the low surgical morbidity associated with thyroid surgery were the second and third most important factors, respectively. The belief in the safety and efficacy of thyroid surgery for treating PTC seems to have a considerable effect on the current general practice favoring total thyroidectomies for patients with PTC of any size. Nonetheless, the currently available guidelines recognize hemithyroidectomy as an appropriate treatment option for low risk PTMC patients [10]¿[12]. Total thyroidectomy carries with it the risk of bilateral recurrent laryngeal nerve injury and higher risks of transient or permanent hypoparathyroidism. While total thyroidectomy has a generally low complication rate, a higher number of this procedure would likely translate into a higher absolute number of complications. In our opinion, decisions around the extent of thyroidectomy should be based on patient and tumor factors and the available evidence/guidelines rather than confidence in the safety for a given procedure.

The preference of the referring physician was the factor identified as least important by respondents. Similarly, American OHNS and general surgeons, in a recent survey, identified the preference of the referring physician as the least important factor in their decisions surrounding the management of PTMC [15]. In that survey multifocality of the disease, ease of follow up post total thyroidectomy, and the need to administer RAI were the three factors identified as most important in influencing the decision to proceed with thyroidectomy.

Our study has some limitations. The response rate was relatively low which may have led to a response bias. Wu *et al.* in a similar study of American Academy of Otolaryngology¿ Head and Neck Surgery (AAOHNS) members achieved a response rate of 3% [15]. physicians and surgeons are known for their poor survey response rates regardless of delivery method [21]. We suspect that low-volume OHNS and endocrinologists and ones with different practice interests differentially did not reply to our survey and therefore this should not affect the generalizability of our results. Our sample size was relatively small for subgroup analysis based on age, practice type, and surgical/clinical volume. Nonetheless, this is one of the largest absolute number of responses reported in the literature addressing this question. Another important issue was that a survey may not accurately reflect true practice patterns. A country wide sampling of charts for assessment of actual management choices would have provided a more accurate reflection of practices, however, self-reported questionnaires offer an estimate of these decisions while being quick and less expensive.

In summary, the current practices of Canadian OHNS and endocrinologists, for the most part, coincide with the available guidelines. The slight variation in recommendations might be explained by the lack of strong evidence addressing the management of PTMC within the guidelines, and the fact that there is contradicting evidence supporting different management options. These controversies need to be explained to the patients in order to allow them to be an integral part of the decision making process. Male gender was the only factor with consistent and significant deviation from the guidelines with both OHNS and endocrinologists recommending a more aggressive treatment option for male patients compared to female patients. Long-term observational survival studies are needed to compare outcomes of PTMC in T1b (1¿2 cm) and T2 (2¿4 cm) nodules. The latest version of the ATA, BTA and ETA address the management of PTMC within the context of PTC. Given the dramatic increase in the incidence of PTMC, it might be prudent to allot PTMC a separate section within updated guidelines.

## Competing interests

All authors declare that they have no competing interests.

## Authors¿ contribution

MM, DG, AS, SE, LR, JF designed the survey questions. MM and DG analyzed the data. MM, AE, DG wrote the manuscript. AS, SE, LR, JF, JD reviewed and revised the manuscript. All authors read and approved the final manuscript.
